# Supplementary material for: Proliferation dynamics of acute myeloid leukaemia and haematopoietic progenitors competing for bone marrow space
Source: Nat Commun. 2018 Feb 6;9:519. doi: 10.1038/s41467-017-02376-5 (PMC5802720; doi:10.1038/s41467-017-02376-5)
Supplement: Supplementary file 1 — Supplementary Information [file 41467_2017_2376_MOESM1_ESM.docx]

**
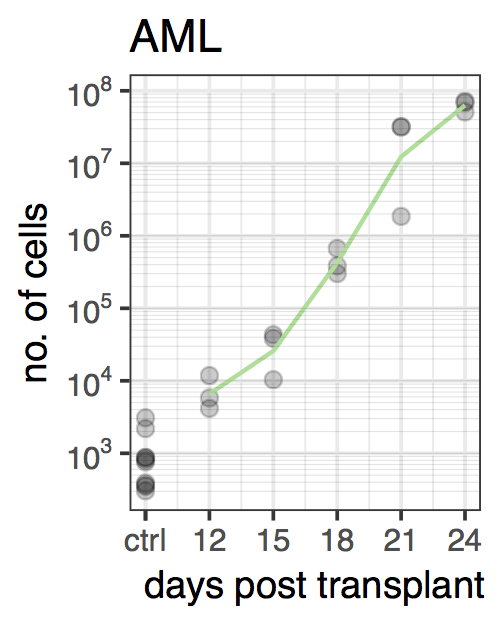
**

**Supplementary Figure 1. AML cell numbers in spleen**. Number of AML cells per spleen are plotted as a function of time (n=10 control mice, n=15 leukaemic mice with 3 analysed per time point).


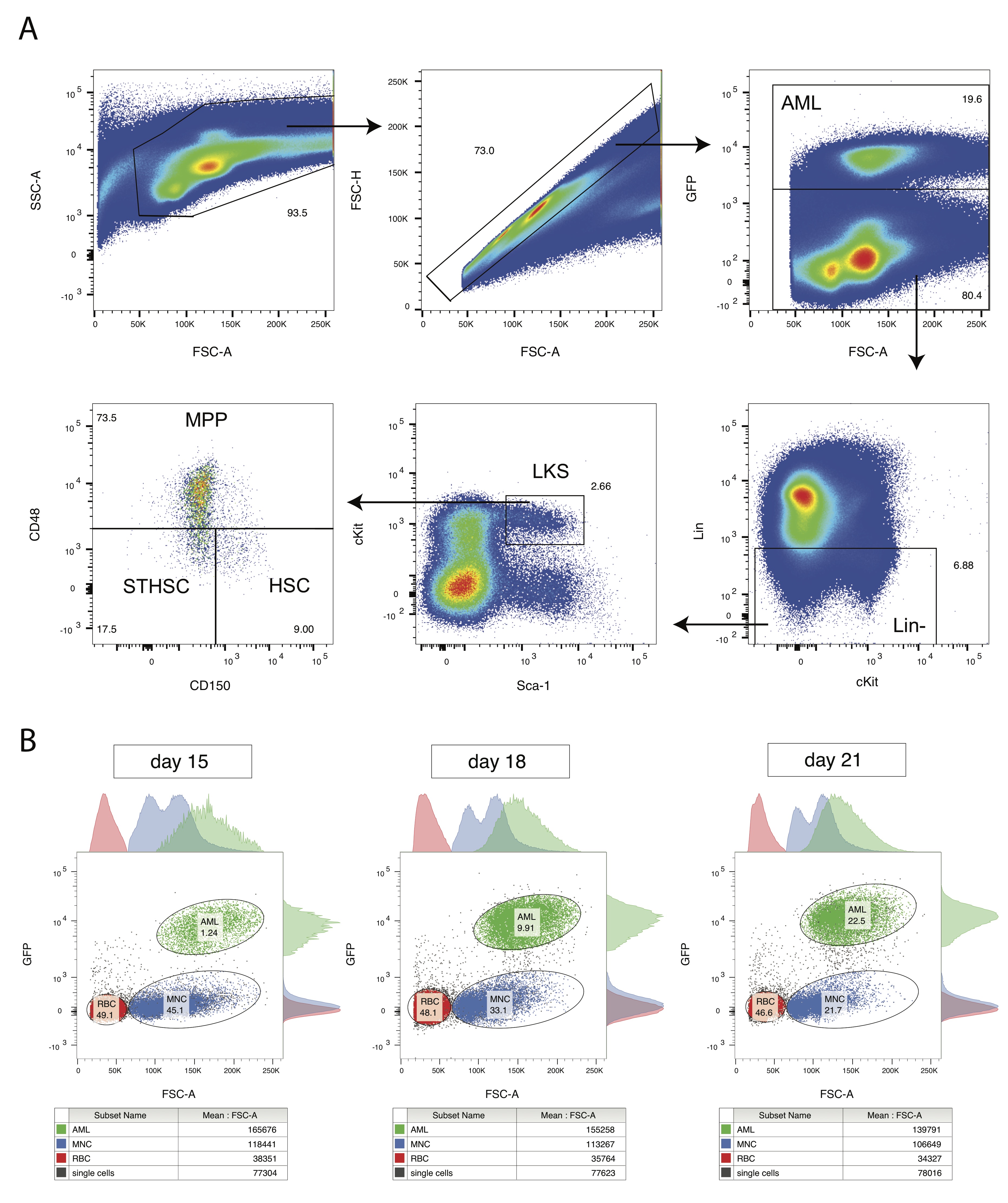


**Supplementary Figure 2. Representative gating strategy, and FSC-A comparison of MNC and AML.** **A.** Representative gates used throughout the manuscript, including gating of AML cells, and gates used to identify primitive haematopoietic populations in the remaining healthy compartment. **B.** FACS plot of GFP vs. FSC-A from a representative AML burdened mice at days 15, 18 and 21, showing AML cells are larger than MNC cells.

**Supplementary Figure 3. Correlation between bone marrow and peripheral blood infiltration by AML cells.** AML BM and PB infiltration was measured at a range of days (colour coded) post-transplant into secondary recipients (n=59 mice, with data pooled from four independent cohorts of mice injected with three distinct AML primary samples). The ratio of PB/BM infiltration is statistically distinct for BM infiltration that is less than or greater to 60% (p=10^-8^, n=35 and n=24, respectively, Kolmogorov-Smirnov two sample test).

**Supplementary Figure 4. Limited apoptosis and death of primitive haematopoietic cell populations.** c-Kit enriched BM cells were stained with annexin V and DAPI to discriminate alive (green), apoptotic (grey) and dead (black) cells (average shown, s.e.m. <1.2% and is not shown; n=3 mice analysed).

**Supplementary Table 1. Antibodies used**

| **Colour** | **Antibody** | **Clone** | **Cat #** | **Supplier** | **Dilution** |
| --- | --- | --- | --- | --- | --- |
| Biotinylated | CD45R/B220 | RA3-6B2 | 103204 | BioLegend | Cocktail used at 1 in 20 |
| Biotinylated | CD3ε | 145-2C11 | 100304 | BioLegend |  |
| Biotinylated | CD4 | GK1.5 | 100404 | BioLegend |  |
| Biotinylated | CD8a | 53-6.7 | 100704 | BioLegend |  |
| Biotinylated | CD11b | M1/70 | 101204 | BioLegend |  |
| Biotinylated | Ly-6G/C (Gr-1) | RB6-8C5 | 108404 | BioLegend |  |
| Biotinylated | Ter-119 | Ter-119 | 116204 | BioLegend |  |
| FITC | CD45.1 | A20 | 110706 | BioLegend | 1 in 100 |
| APC/Cy7 | CD117 (c-Kit) | 2B8 | 105826 | BioLegend | 1 in 100 |
| PerCP/Cy5.5 | Ly-6A/E (Sca-1) | D7 | 108124 | BioLegend | 1 in 100 |
| PE/Cy7 | CD48 | HM48-1 | 103424 | BioLegend | 1 in 100 |
| BV 650 | CD150 | TC15-12F12.2 | 115931 | BioLegend | 1 in 100 |
| FiTC | CD45.1 | A20 | 110706 | BioLegend | 1 in 100 |
| PE/Cy7 | CD16/32 | 93 | 101318 | BioLegend | 1 in 100 |
| PE | CD34 | HM34 | 128610 | BioLegend | 1 in 100 |
| BV711 | CD127 (IL-7Rα) | A7R34 | 135035 | BioLegend | 1 in 100 |
| Alexa Fluor 647 | BrdU | MoBU-1 | B35140 | Life Technologies | 6.5 in 100 |
| Pac Orange | Streptavidin |  | S32365 | Life Technologies | 1 in 1000 |

This table lists all antibodies used in this study, including their conjugated fluorophore, clone, supplier, catalogue number and dilution used.
